# Supplementary material for: Decoding the protein–ligand interactions using parallel graph neural networks
Source: Sci Rep. 2022 May 10;12:7624. doi: 10.1038/s41598-022-10418-2 (PMC9086424; doi:10.1038/s41598-022-10418-2)
Supplement: Supplementary file 1 — Supplementary Information. [file 41598_2022_10418_MOESM1_ESM.pdf]

Supporting Information: Decoding the Protein-ligand Interactions Using Parallel Graph Neural Networks

Carter Knutson [‖], Mridula Bontha [‖], Jenna A. Bilbrey [‖], and Neeraj Kumar[‖*]

[‖] *Pacific Northwest National Laboratory, 902 Battelle Blvd, Richland, WA 99352, United States*

E-mail: neeraj.kumar@pnnl.gov

Phone: +1 509-372-6422

Layer Summary

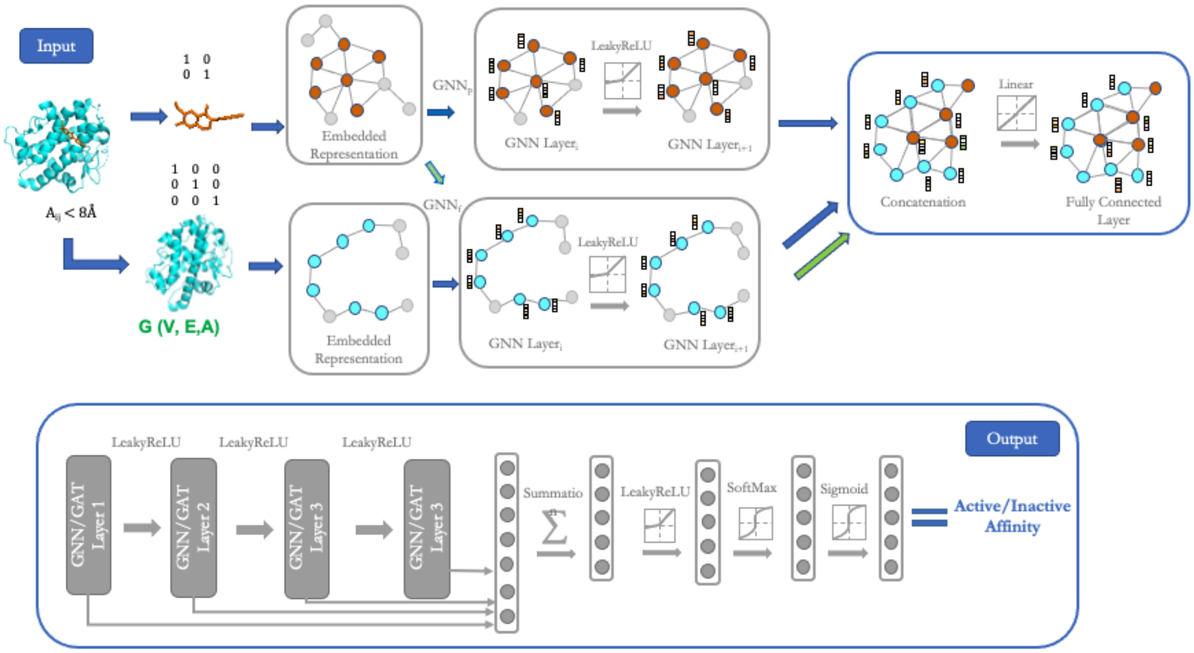

Figure S1: In depth layer summary of the GNN_F(denoted with a green arrow) and GNN_P models. Embedded representations of the protein and ligand feed into each model where they are combined in the GNN_F and processed separately in the GNN_P. The base layer logic is the same for each model, outlined in blue, the only difference is the complex representation.

Protein Similarity Analysis

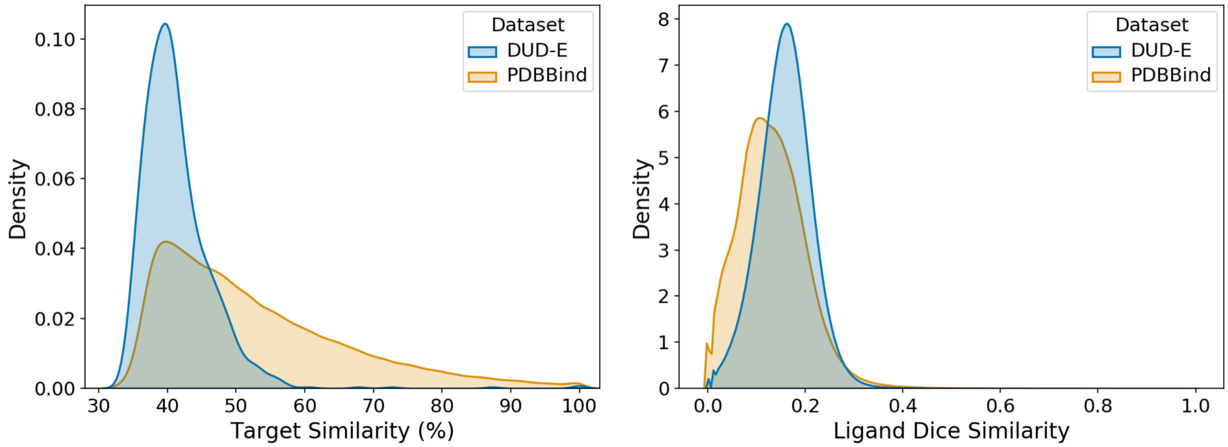

Figure S2: Similarity of protein targets in the DUD-E and PDBbind datasets based on the homology of each target pair, and Dice similarity of the Morgan fingerprints of ligands in the DUD-E and PDBbind datasets. Because the DUD-E dataset contains a large number of ligands, not all pairs could be analyzed, so 10,000 ligands were chosen at random from the dataset for analysis.

Hyperparameter Optimization

Trainings were carried out over 200 epochs on a quarter of the data taken from our dataset consisting of 2,000 samples per target with 79 targets, using the same train-test split for all trainings. We used the parameters from our best model to create a baseline: a learning rate of 0.0001, four attention heads, and a layer dimension of 70 produced an average test AUROC of 0.854. The decreased performance compared to the models discussed in the Results & Discussion section is expected due to the reduced set of data used to train the model. Thirty of the 36 combinations trained without error.

Table S1: Values of examined hyperparameters: learning rate (lr), number (N) of attention heads, and dimension (D) of the GAT layer in each attention head. A grid search was performed on each combination of parameters. The optimal combination is shown in bold.

lr	N	D
0.001	**2**	**70**
**0.0001**	3	140
0.00001	4	210
		280

Table S2: Values of examined hyperparameter set with average train and Test ROC .

HYPERPARAMETER SET	TRAIN ROC AVG	TEST ROC AVG
LR_0.001_N5_D70	0.754	0.771
LR_0.001_N4_D210	0.606	0.647
LR_0.001_N4_D140	0.605	0.661
LR_0.001_N3_D70	0.834	0.835
LR_0.001_N3_D280	0.610	0.630
LR_0.001_N3_D210	0.672	0.694
LR_0.001_N3_D140	0.714	0.742
LR_0.001_N2_D70	0.937	0.861
LR_0.001_N2_D140	0.788	0.781
LR_0.001_N2_D280	0.762	0.755
LR_0.001_N2_D210	0.767	0.773
LR_0.0001_N4_D128	0.898	0.854
LR_0.0001_N3_D70	0.894	0.855
LR_0.0001_N3_D210	0.930	0.860
LR_0.0001_N3_D140	0.917	0.862
LR_0.0001_N2_D70	0.912	0.864
LR_0.0001_N2_D210	0.950	0.861
LR_0.0001_N2_D140	0.940	0.853
LR_0.00001_N4_D70	0.707	0.726
LR_0.00001_N4_D210	0.776	0.783
LR_0.00001_N4_D140	0.748	0.769
LR_0.00001_N3_D70	0.735	0.758
LR_0.00001_N3_D210	0.778	0.772
LR_0.00001_N3_D140	0.763	0.755
LR_0.00001_N2_D70	0.713	0.749
LR_0.00001_N2_D210	0.776	0.783
LR_0.00001_N2_D140	0.774	0.782

Performance Metrics for Classification and Regression GNN Models

Table S3: Accuracy of each experiment evalutating the affects of additional DUD-E targets as well as sample consideration per target for both the GNN_F and GNN_P models.

MODEL	TARGET	SAMPLES	ACC
GNN_F	17	1k	0.764
GNN_F	79	1k	0.888
GNN_F	96	1k	0.851
GNN_F	17	2k	0.764
GNN_F	79	2k	0.912
GNN_F	96	2k	0.900
GNN_P	17	1k	0.686
GNN_P	79	1k	0.837
GNN_P	96	1k	0.791
GNN_P	17	2k	0.681
GNN_P	79	2k	0.880
GNN_P	96	2k	0.828

Table S4: Performance comparison of our GNN models in predicting Experimental affinity on PDBbind2016 core set. (*Cases where the test set contains more than just the core set)

	**Just core set**				**Entire test set**			
**Model**	**RMSE**	**MAE**	**Pearson r**	**Spearman r**	**RMSE**	**MAE**	**Pearson r**	**Spearman r**
GNN_F-EBA*	1.73	1.42	0.65	0.63	1.61	1.28	0.58	0.57
**GNN_P-EBA***	**1.73**	**1.39**	**0.62**	**0.62**	**1.55**	**1.21**	**0.58**	**0.59**
Pafnucy[?]	1.42	1.13	0.78	-	-	-	-	-
SG-CNN (R + G)[?]	1.37	1.08	0.78	0.76	-	-	-	-
3D-CNN (R + G)[?]	1.68	1.33	0.67	0.65	-	-	-	-
midlevel fusion[?]	1.308	1.019	0.810	0.807	-	-	-	-
**K_{DEEP}[?]**	**1.27**	**-**	**0.82**	**0.82**	**-**	**-**	**-**	**-**

PDBbind2016 dataset split

Table S5: Table summarizing the PDBbind2016 dataset split used for Experimental Affinity regression models.

Dataset	Total samples	Train samples	Test samples
PDBbind2016-EBA:75:25	11,674	8,248	3,136
PDBbind2016-EBA:80:20	11,674	8,808	2,276
PDBbind2016-EBA:90:10	11,674	10,246	1,428
PDBbind2016-EBA:core	11,674	11,384	290

The PDBbind2016-EBA core dataset consists of the entire general and refined set as the train set and just the core set as the test set. The rest of the model's test set includes a fraction of the general and refined dataset along with the core set.

GNN_F and GNN_P 1k and 2k Comparison

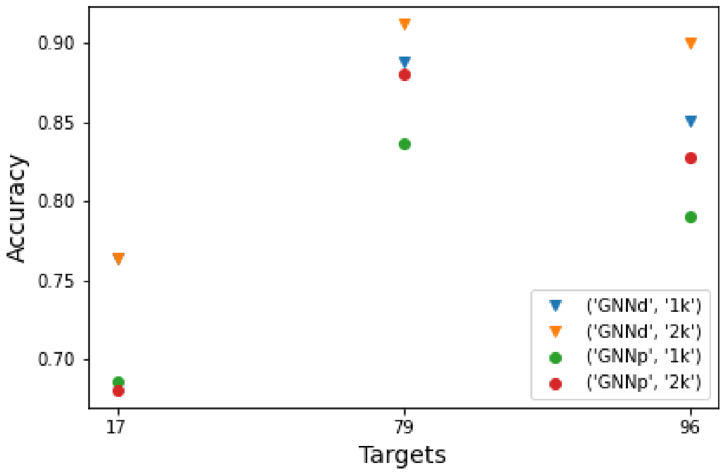

Figure S3: Dataset performance comparisons of the GNN_F and GNN_P models. Datasets composed of 17,79, and 96 DUD-E targets, all available PDBbind data, and implementations of 1,000 and 2,000 samples per DUD-E target. This shows how increasing the amount of targets included in the sample set, and the amount of individual samples affect model performance.

Top-N Ranks Analysis of Classification Models

We compare the performance of GNN to docking in identifying protein-ligand complexes in top-n ranks. We choose the best GNN model (2k_79) to get predictions on the train and the test dataset. For the GNN, we rank the models in ascending order of predicted binding probabilities. For docking we rank the models based on the descending order of binding affinity scores obtained as a part of docking pipeline. We assess the performance of each of the scoring method (GNN and docking) on their ability to identify protein-ligand models with RMSD less than 2Å from the experimental crystal structure. Because the crystal structure is only available for the PDBbind data, we preformed the analysis on only the PDBbind dataset, once on the test designated targets, and once over the entire set. Here we report the percentage of targets identified which had at least one model identified in top-N ranks when ranked according to the GNN and Docking scores. Here, we are evaluating on 676 targets from training and 159 targets from test which were common for all three scoring methods. From the graph we can see that GNN_F and docking preformed nearly identically on training data while the GNN_P preformed poorly. The performance on the test data is same for all the methods.

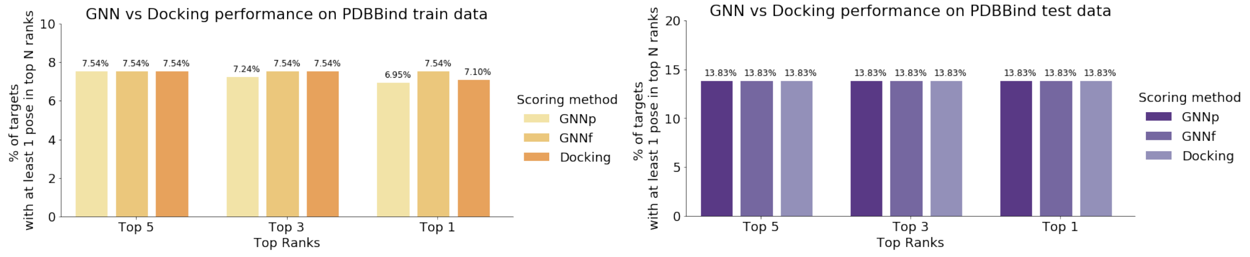

Figure S4: Comparision of GNN_p and GNN_f model with Docking. Each bar corresponds to the percentage of protein-ligand complexes with at least 1 docked pose in Top-N ranks which have an RMSD less than 2Å from its crystal structure.

To measure the potency of GNN models we assess the model's ability to identify docked poses per protein-ligand complex. With this, we not only measure the ability of the model to identify an active protein-ligand pose but also its ability to identify the best pose amongst

multiple docked calculations for a protein-ligand complex (see Fig. SS4).

IBS molecule properties

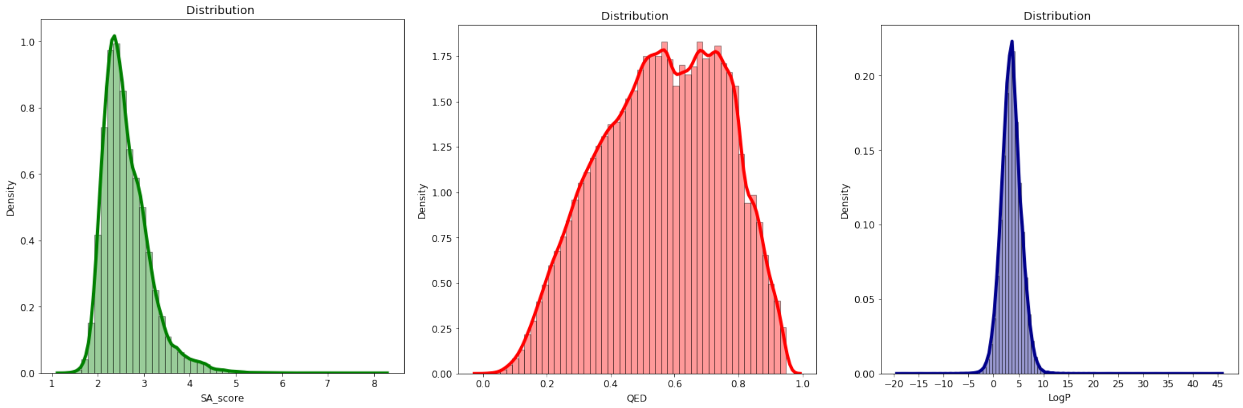

Figure S5: Distribution of properties of IBS molecules to measure their drug-likeliness. The properties include the synthetic accessibility (SA) score, quantitative estimation of drug-likeliness (QED), and the partition coefficient (logP).

The SA score is the synthesizability of generated molecules and ranges between 0-10, where the lower end suggests increased accessibility. QED is the quantifying and ranking of the drug-likeness of a compound. The values range from 0 for unfavorable to 1 for favorable. The partition coefficient, logP is a measure that determines the physical nature of a compound and its ability to reach the target in the body. A positive logP value indicates the compound is lipophilic and a negative logP value indicates a hydrophilic compound. We can see that more than 60% of compounds have a low SA score (indicates easily synthesizable) and a high QED (indicates high drug-likeliness) and a lopP between -0.4 and 5.6. These properties suggest that considerably a greater number of IBS molecules could have a high potency to bind to a known receptor.

IBS molecules - Binding probability vs molecular weight

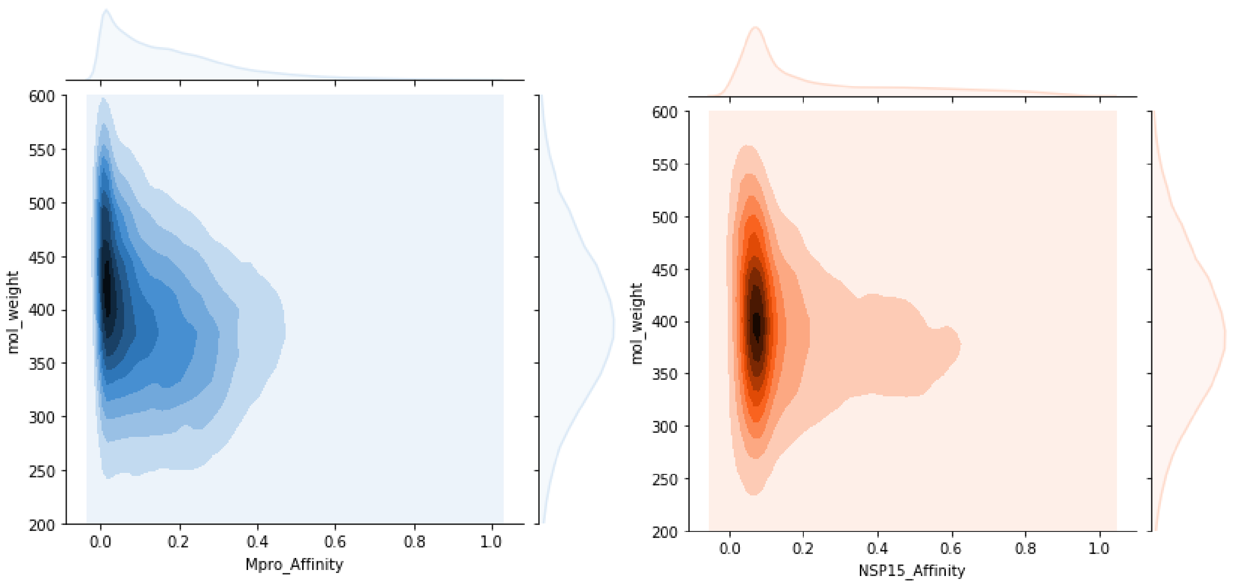

Figure S6: Density plot for molecular weight vs binding probability for IBS molecules.

pIC_{50} of Active Targets and Predicted Binding Affinity Relationship Analysis

In this section we discuss the IC_{50} activity of the DUD-E active targets and their correlation with the predicted binding probability. We chose the predicted binding probability of the model with the lowest docking energy as we had multiple poses for each molecule obtained through the docking pipeline. We found that majority of the molecules have pIC_{50} between 10 and 20. The expected trend here is to have high binding probability associated with a high pIC_{50} value.

The GNN_F model displays increased performance to the GNN_P as the density of higher pIC_{50} valued molecules is concentrated at high binding probability values, as shown in Figure S7. We can see a similar trend for the GNN_P model as well, except that the model identifies a majority of high pIC_{50} valued compounds as poorly binding complexes. While looking at the performance on the training dataset, both GNN_F and GNN_P show good learning

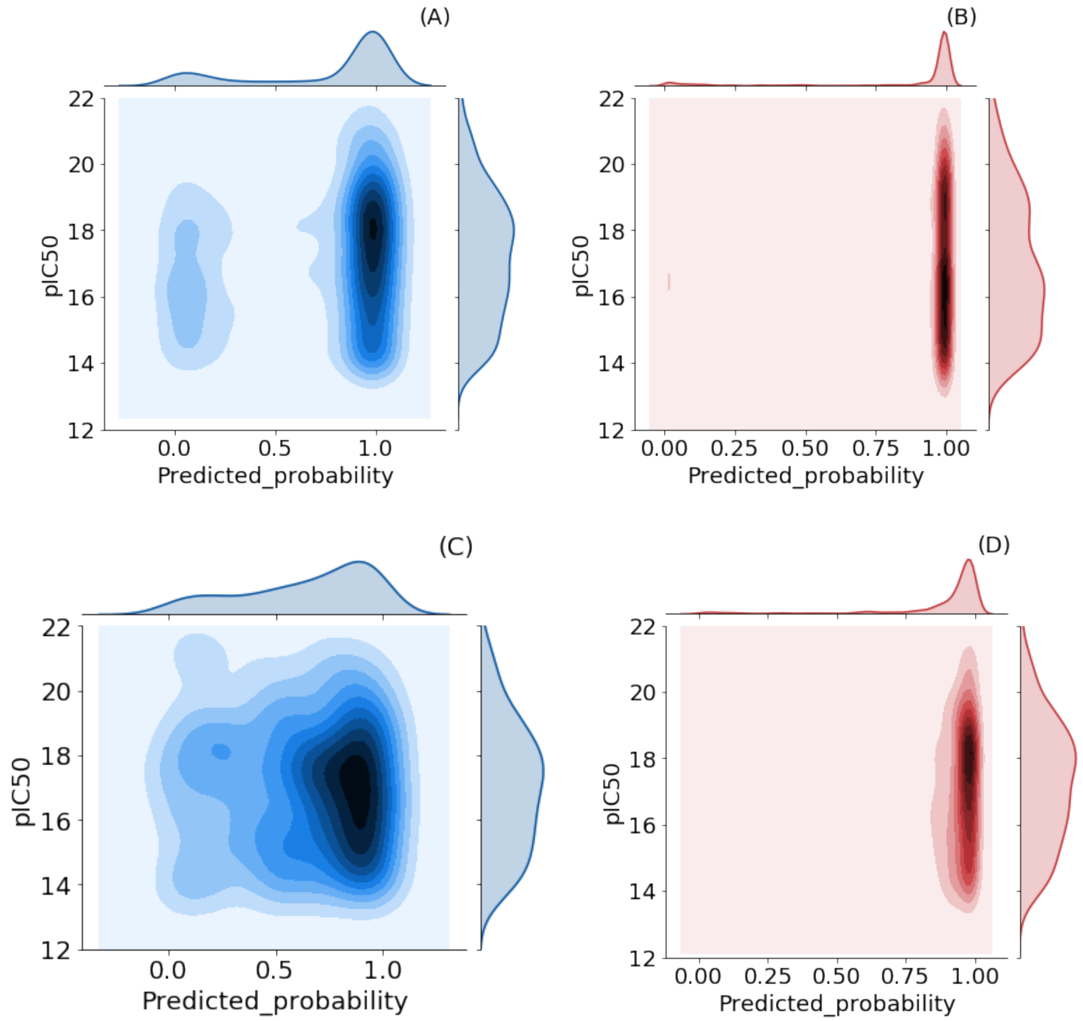

Figure S7: pIC_{50} vs Binding probability for DUD-E active molecules. Figure (A) and (B) correspond to test and train sets for GNN_F and figure (C) and (D) correspond to the GNN_P model. The model was able to associate high binding probabilities to high pIC_{50} valued molecules.

by attributing high pIC_{50} compounds with a high binding probability. Overall we can say that GNN_F has shown a good performance on both training and test set while learning the correlation between the experimental binding affinity and probability of binding.

The GNN_p test set has a bimodal distribution for probability 0, so it gave errors on a number of low IC_{50} samples compared to GNN_f where the errors were generally associated with high IC_{50} samples.

GNN vs FAST Model Comparison

Table S6: Performance comparison of our GNN models and FAST models on the same data split from PDBbind2016. While the SG-CNN shows the best performance on the R + G split and 3D-CNN gives best performance on the refined set, we achieve the best performance using the GNN_P model on the general set.

MODEL	RMSE	MAE	Pearson r	Spearman r	r^2
GNN_F-EBA (R)	1.92	1.56	0.51	0.50	0.26
GNN_F-EBA (G)	1.84	1.48	0.58	0.59	0.34
GNN_F-EBA-(R + G)	1.75	1.42	0.61	0.60	0.37
GNN_P-EBA (R)	1.72	1.37	0.63	0.62	0.40
**GNN_P-EBA (G)**	**1.66**	**1.33**	**0.66**	**0.67**	**0.43**
GNN_P-EBA-(R + G)	1.69	1.33	0.64	0.66	0.41
SG-CNN (R)	1.65	1.32	0.66	0.64	0.42
SG-CNN (G)	1.50	1.19	0.74	0.74	0.51
**SG-CNN (R + G)**	**1.37**	**1.08**	**0.78**	**0.76**	**0.60**
3D-CNN (R)	1.5	1.16	0.72	0.71	0.52
3D-CNN (G)	1.65	1.29	0.64	0.65	0.42
3D-CNN (R + G)	1.68	1.33	0.67	0.65	0.39

Docking Binding affinity Data and Results

For Docking Binding Affinity (DBA) regression models, we used 1000 positive and 1000 negative poses per target in the DUD-E dataset as we did for the classification dataset. In addition, for the DBA scores, we used the docking score as obtained from the docking pipeline for all the PDBbind and DUD-E decoys.

Table S7: Table summarizing the dataset used for Docking Binding Affinity (DBA) regression models.

Dataset	Total targets	Total ligands	Train targets	Test targets	Train samples	Test samples	Total samples
PDBbind2018-DBA	991	991	867	124	5,410	812	6,222
DUD-E-DBA	65	5,358	54	11	105,814	22,218	128,032

Table S8: Performance comparison of our GNN models in predicting Docking affinity on test dataset.

MODEL	RMSE	MAE	Pearson r	Spearman r	r^2
$\mathrm{GNN_F}$-DBA	0.80	0.63	0.78	0.79	0.62
$\mathrm{GNN_P}$-DBA	0.84	0.66	0.72	0.70	0.51
